# Clinical study of the effect of mometasone furoate nasal spray treatment on hearing and in secretory otitis media in children

**DOI:** 10.1016/j.clinsp.2024.100551

**Published:** 2024-12-12

**Authors:** Xiaoyan Yu, Lang Xu, Youqi Xie, Mengjie Huang

**Affiliations:** Department of Otolaryngology, Chengdu Women's and Children's Central Hospital, (The Affiliated Women's and Children's Hospital, School of Medicine, UESTC), Chengdu City, Sichuan Province, PR China

**Keywords:** Mometasone furoate, Secretory otitis media, Younger children, Hearing, Inflammatory response

## Abstract

**Objective:**

To investigate the effect of Mometasone furoate (Elocon Cream) Nasal Spray (MFNS) treatment on hearing secretory Otitis Media (SOM) in younger children.

**Methods:**

Seventy-six children with SOM (ages 5 to 10 years-old) were selected as study subjects and divided into two groups of 38 cases each using a randomized numerical table. The control group was given conventional treatment, and the observation group was treated with MFNS based on the control group. Both groups were treated for 12 weeks. The improvements in clinical symptoms and hearing were compared between the two groups at weeks 4, 8 and 12 of treatment, respectively.

**Results:**

The total effective rate of treatment in the observation group was higher than that in the control group. The middle ear resonance frequency of the children in the observation group was higher than that of the control group at weeks 4, 8 and 12 of treatment, and the air-conduction hearing threshold was lower than that of the control group. The total effective rate of the observation group after 12-weeks of treatment was 92.11 %, which was significantly higher than that of the control group (73.68 %). In addition, the T-ETDQ score of the observation group was lower than that of the control group after treatment.

**Conclusion:**

MFNS has clinical efficacy in the treatment of SOM in young children, which can improve clinical symptoms, promote the recovery of hearing and eustachian tube function, reduce the local inflammatory response, and improve immune function.

## Highlights

• The total effective rate of treatment in the observation group was higher than that in the control group.
• The middle ear resonance frequency of the children in the observation group was higher than that of the control group at weeks 4, 8 and 12 of treatment, and the air-conduction hearing threshold was lower than that of the control group.
• The total effective rate of the observation group after 12 weeks of treatment was 92.11 %, which was significantly higher than that of the control group (73.68 %).
• The T-ETDQ score of the observation group was lower than that of the control group after treatment.

## Introduction

Secretory Otitis Media (SOM) is a common and frequent disease in otology, mostly occurring in winter and spring, and is one of the common causes of hearing impairment in children and adults. .^1^ Clinical diagnosis and treatment of SOM are often delayed due to the relatively mild symptoms of ear pain and hearing loss at the onset of SOM, especially in children, which can lead to permanent hearing loss and speech developmental disorders if not diagnosed and treated in a timely manner.^2^ Therefore, this disease should be treated with high vigilance and sufficient attention in clinical practice. Oral medication is the main means of treatment for SOM, including antibiotics, glucocorticosteroids, and mucus promoters. However, the efficacy of oral medication alone is often unsatisfactory.^3^^,^^4^

mometasone furoate (Elocon Cream) Nasal Spray (MFNS) is a potent glucocorticoid with strong anti-inflammatory properties, which inhibits cellular response and reduces inflammation-related mediator synthesis and release. Clinical data show that MFNS can eliminate mucosal swelling and reduce inflammation and swelling of the nasal cavity and nasopharynx caused by the mechanical compression of the Eustachian tube, prompting the opening of the Eustachian tube to reduce the negative pressure in the middle ear cavity, so that the middle ear exudate is reduced or discharged.^5^ However, there are few clinical reports on its therapeutic effects on younger children with low-grade SOM.

Based on this, this study aims to investigate the effect of MFNS treatment on hearing and local inflammatory response of SOM in younger children, so as to provide a reference basis for the clinical treatment of younger children with this disease.

## Materials and methods

### Clinical data

Seventy-six children with SOM from January 2021 to June 2023 were studied, and they were divided into two groups using the randomized numerical table method, with 38 cases/group. Inclusion criteria (1) Children meeting the diagnostic criteria of SO;M^6^ (2) Children's family members gave informed consent and signed the consent form; (3) Children with clinical symptoms such as stuffy ear, hearing loss, tinnitus, etc.; (4) Children with intact tympanic membrane; (5) Otoscopic examination suggests that tympanic membrane is inverted and fluid is accumulated in the middle ear; and (6) Age between 5‒10 years-old.

Exclusion criteria: (1) Children with neural hearing loss; (2) Children with symptoms of acute infection of the upper respiratory tract or the ear; (3) Children with anatomical abnormalities of the ostiomeatalex; (4) Children with indications for surgery for adenoid hypertrophy; (5) Children with nasalpolyps; (6) Children with primary immune deficiencies; (7) Children with primary ciliary dyskinesia; (8) Children with congenital abnormalities of the airway and lung parenchyma; (9) children with allergies to mometasone furoate (Elocon Cream), amoxicillin, Ambroxol Hydrochloride, and Methylprednislolne tablets. The present study was approved by the Ethics Committee of Chengdu Women's and Children's Central Hospital and written informed consent was provided by all patients prior to the study start (approval number: 202003CD28). And the study follows the STROBE statement.

### Treatment methods

The control group was given conventional oral drug treatment, including Clarithromycin Dispersible Tablets (0.125 g, 2 times/day); Ambroxol Hydrochloride tablets (15 mg, 3 times/day); Methylprednislolne tablets (4 mg, 1 time/day) for two weeks. The local nasal cavity was treated with 1 % Ephedrine Hydrochloride drops (2 drops each time, twice a day) for 2 weeks. Sterile saline (100 mL) at 37 °C was used to rinse the nasal cavity. Irrigation method: Before irrigation, 1 % Ephedrine Hydrochloride drops were administered into the nasal cavity bilaterally twice. With the child in a supine head-down position, the rinse solution was dropped into the nasal cavity for repeated rinsing, and under continuous negative pressure, each side of the nasal cavity was rinsed 3 to 5 times until the nasal cavity was clear without secret retention. Rinsing was performed 2 times a day for 2 weeks. In addition, nasal saline spray was needed twice daily for 8 weeks. This was followed by 1 nasal saline spray per day for 4 weeks. The total treatment cycle was 12 weeks.

In the observation group, nasal saline spray was replaced with MFNS in the control group, one spray per nostril (50 μg/spray) twice daily (4 sprays in total) for 8 weeks, with 200 μg/day for the first 8 weeks. Subsequently, one spray (2 sprays total) was administered once daily for 4 weeks.

### Observation indices

Clinical treatment effect determination criteri:a^7^ the two groups were subjected to Pure Tone Audiometry (PTA) and tympanometry testing after 12 weeks of treatment, and the improvement of symptoms and tympanic membrane conditions were recorded for the evaluation of efficacy. Efficacy evaluation criteria: Cured: deafness, ear stuffiness, and blockage disappeared, air-conducted hearing threshold reduced to 0‒25 dB HL, tympanogram was A type, and tympanic membrane morphology was normal; Improved: deafness, ear stuffiness, and blockage were reduced, air-conducted hearing threshold was reduced, but it did not reach 25 dB HL, the tympanogram was C type or changed from C to As type, and the tympanic membrane morphology was basically normal or inverted; Ineffective: no improvement in clinical symptoms and various examinations. Classification of each type of tympanic conductance ma:p^8^ Type A: normal tympanogram with a peak of −100 to +50 daPa and a peak amplitude of 0.3 to 1.67 mL; Type B: no peak or peak amplitude of < 0.3 mL; Type C: a normal tympanogram but with a negative pressure of more than −100 daPa; and Type As: normal tympanogram with a peak of 0 daPa and a peak amplitude of < 0.3 mL.

Improvement of clinical symptoms: The disappearance time of tinnitus and ear stuffiness, hearing recovery time, effusion persistence time, and tympanic membrane healing time were compared between the two groups at weeks of 4, 8, and 12.

Hearing: Under the condition of 500∼2000 Hz pure tone, air-conducted and bone-conducted hearing thresholds and middle-ear resonance frequency were recorded using ITERA Ⅱ pure tone audiometer (Shanghai Thermo Biotechnology, Shanghai, China) before treatment and at weeks 4, 8 and 12 post-treatment.

Tubomanometry (TMM): measurements were performed using an Eustachian tube manometer (Tubomanometer, Germany). During the test, the child placed a suitably sized earplug in the opening of the external auditory canal and kept it sealed. The child drunk water in the mouth. The tester selected a suitable nasal adapter to block both sides of the patient's nasal cavity and keep it sealed. The pressure was adjusted to 30, 40 and 50 mbar. After blocking the vent, the patient was forced to swallow. Pressure changes in the nasopharynx and external auditory canal were recorded at three different pressures using pressure transducers in the nasopharynx and external auditory canal. Point C1 indicates the onset of pressure increase in the nasopharynx, point C2 indicates the maximum increase in the nasopharyngeal pressure, point P1 indicates the onset of pressure increase in the external auditory canal, and point P2 indicates the maximum increase in pressure in the external auditory canal. The opening latency index (R) of the Eustachian tube was calculated according to the formula *R* = (P1-C1)/(C2-C1). Where *R* ≤ 1 indicates normal opening of the Eustachian tube, *R* > 1 indicates delayed opening of the Eustachian tube, an undetected value of R indicates obstruction of the Eustachian tube, and *R* = 0 indicates abnormal opening of the Eustachian tube.^9^ Delayed opening or obstruction of the eustachian tube indicates abnormal eustachian tube function, and those with abnormal eustachian tube opening were not included in this study.

Eustachian tube function: Eustachian Tube Dysfunction Questionnaire (ETDQ-7) score and Eustachian Tube Score (ETS) were used to evaluate the Eustachian tube function before treatment and after 12-weeks of treatment.^10^^,^^11^ The ETDQ-7 score mainly consists of seven items including ear stuffiness, ear pain, and ear obstruction or pressure, with a score of 0∼7 for each item, and a total score of ≥ 14.5 (Or ≥ 2.1 average score for each item) indicated that the patients had Eustachian tube dysfunction. The ETS score mainly combines objective evaluation and subjective evaluation, and a score of < 5 indicates Eustachian tube dysfunction.

T-ETDQ scores were assessed before and after 12-weeks of treatment. It consists of the TMM and the ETDQ-7 scale. A score of 0 was recorded when *R* ≤ 1 was detected by the TMM; a score of 1 was recorded when *R* > 1; a score of 2 was recorded when no R value was detected; and scores measured at three pressures (i.e., 30, 40, and 50 mbar) were summed up to a maximum score of 6. The mean score of ETDQ-7 indicates subjective scoring. The sum of the subjective and objective scoring results is the T-ETDQ score, and the maximum score is 13. The higher the score, the more severe the Eustachian tube dysfunction.

### Statistical analysis

The data obtained were processed by SPSS 22.0 software. Enumeration data were expressed as % and comparatively assessed by χ^2^ test. Measurement data were expressed as mean ± standard deviation after a normality test and compared by *t*-test; *p* < 0.05 means the difference was statistically significant.

## Results

### Clinical data

Clinical data from the two groups did not show any significant differences (*p* > 0.05, Table 1).

**Table 1:** Comparison of clinical data between the two groups.

Factors	Observation group (*n* = 38)	Control group (*n* = 38)	χ2/t	p
Gender (cases)			0.211	0.646
Male	21	19		
Female	17	19		
Age (years)	5.08 ± 0.55	5.12 ± 0.47	0.286	0.776
Course of disease (days)	7.96 ± 1.13	8.04 ± 1.09	0.268	0.79
Height (cm)	113.02 ± 11.59	112.94 ± 10.64	0.027	0.979
Body weight (kg)	18.25 ± 2.37	18.63 ± 2.41	0.595	0.554

### Clinical efficacy

The total effective rate of treatment in the observation group was higher than that in the control group (*p* < 0.05, Table 2).

**Table 2:** Comparison of clinical efficacy between the two groups (cases, %).

Groups	n	Cured	Improved	Ineffective	Total effective rate
Observation group	38	12	23	3	92.11
Control group	38	9	19	10	73.68
*χ*^2^					4.547
p					0.033

### Improvement of clinical symptoms

**Fig. 1:**
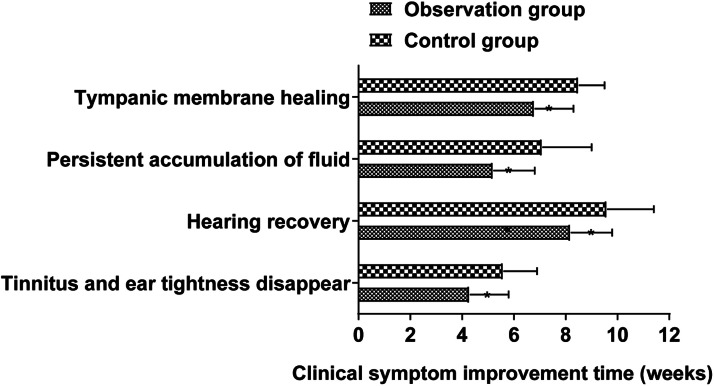
Comparison of the improvement of clinical symptoms between the two groups. (Note: Compared with the observation group, * *p* < 0.05).

The disappearance time of tinnitus and ear stuffiness, hearing recovery time, effusion persistence time, and tympanic membrane healing time of the observation group were shorter than those of the control group (*p* < 0.05, Fig. 1).

### Hearing and eustachian tube function score

**Fig. 2:**
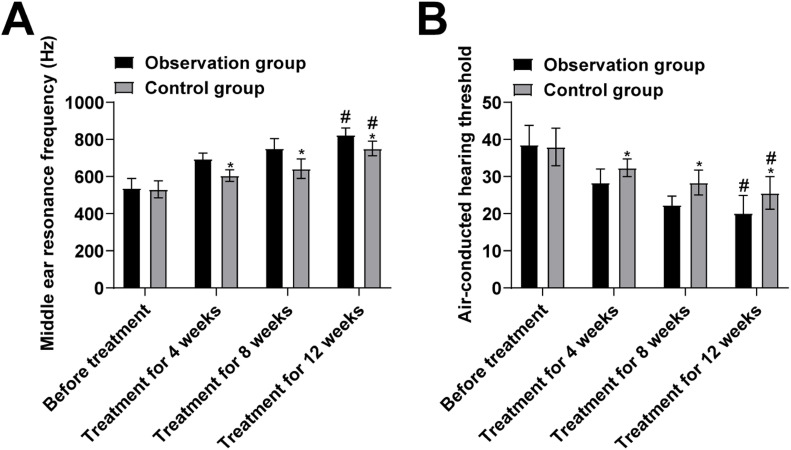
Comparison of hearing between the two groups. (A) Middle ear resonance frequency; (B) Air-conducted hearing threshold. (Note: compared with the pre-treatment, # *p* < 0.05; compared with the observation group, * *p* < 0.05).

There was no significant difference in the comparison of middle ear resonance frequency and air-conducted hearing thresholds between the two groups before treatment (*p* > 0.05, Fig. 2A). In addition, at weeks 4, 8 and 12 of treatment, middle ear resonance frequency was higher after treatment than that before treatment in the two groups, and that in the observation group was higher than the control group (*p* < 0.05, Fig. 2A). After 12 weeks of treatment, air-conducted hearing thresholds were lower than those before treatment in the two groups and were lower in the observation group than of the control group (*p* < 0.05, Fig. 2B).

### Eustachian tube function

**Fig. 3:**
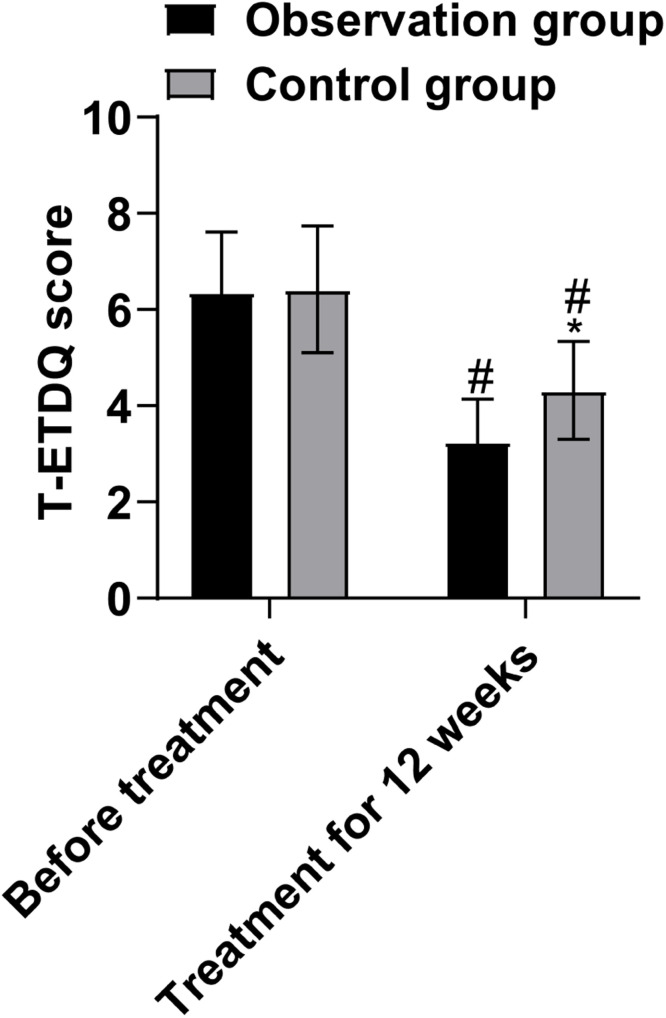
Comparison of Eustachian tube function scores between the two groups (Note: compared with before treatment, # *p* < 0.05; compared with the observation group after treatment, * *p* < 0.05).

Prior to treatment, T-ETDQ scores did not differ significantly between the two groups (*p* > 0.05). After 12 weeks of treatment, T-ETDQ scores were lower than those before treatment, and those of the observation group were lower than the control group (*p* < 0.05, Fig. 3).

### Adverse reactions

No significant adverse reactions were observed during the treatment of children in both groups.

## Discussion

Clinically, SOM is a high incidence otolaryngologic disease, with impaired hearing and middle ear effusion as the main clinical manifestations of this disease.^12^ Children have a high incidence of SOM because the anatomy of the nasopharynx and the immune system are not yet mature. Clarithromycin and other conventional treatments are mostly used for clinical treatment, but the clinical efficacy is not satisfactory.^13^ Relevant studies have pointed out that in the treatment of SOM in children on a traditional basis, the addition of glucocorticoid can reduce inflammation and allergic reactions in the middle ear, Eustachian tube, nasopharynx, and other parts of the body.^14^^,^^15^ MFNS is a potent glucocorticoid with strong anti-inflammatory effects, which can effectively inhibit cellular responses and reduce the synthesis and release of inflammatory mediators. In this study, the authors found that the clinical efficacy of MFNS in the treatment of SOM in younger children is high, which can improve the clinical symptoms of the children and promote the recovery of hearing, mainly because the application of MFNS not only reduces the inflammatory reaction of the nasal cavity and improves the ventilation, but also promotes the synthesis of intracellular anti-inflammatory proteins and reduces the impact on the function of the Eustachian tube, which can then promote the recovery of hearing.^16^

It is currently believed that the pathogenesis of SOM is generally considered to be closely related to Eustachian tube dysfunction and immunologic and infectious factors. Mechanical obstruction of the Eustachian tube affects the pressure difference between the middle ear and the outside world, leading to auditory filling, unilateral hearing loss, and otitis media. PTA is a commonly used clinical technique for the diagnosis of hearing loss.^17^ Studies have shown that PTA can guide surgical decisions in chronic otitis media. The relationship between preoperative PTA and middle ear status helps the surgeon to plan the surgery and inform the patient about the hearing outcome.^18^ The results of this study showed that under 500‒2000 Hz pure tone conditions, air-conducted hearing thresholds and middle ear resonance frequency in the observation group were significantly improved at weeks 4, 8, and 12 of treatment, and the improvement was stronger than that of the control group.

Due to the location of the Eustachian tube, this anatomical location was not easily accessible to otolaryngology in the past. Current studies have shown that the Eustachian tube is correlated with the pathophysiology and treatment of snoring, sinusitis, and SOM in children.^19^ In pediatric patients, anatomical differences in the Eustachian tube, including a shorter, flatter, and wider structure compared to adults, as well as weaker muscle contraction and less elastic cartilage, contribute to a propensity for collapse when the tympanic chamber is under negative pressure. Additionally, underdeveloped masticatory function in children and lack of exercise in the pharyngeal muscles further impact the functionality and development of the Eustachian tube. Therefore, nasopharyngeal obstruction and nasal stenosis are more likely to cause SOM in children. However, previous treatments of chronic SOM fail to improve the function of the Eustachian tube.^20^ Localized nasal and nasopharyngeal treatment is important for improving nasal ventilation and controlling nasal inflammation, which can effectively eliminate the effects of nasal obstruction and inflammation on the Eustachian tube, and is conducive to promoting the recovery of Eustachian tube function.^21^ Nasal irrigation is one of the therapeutic means for the treatment of rhinitis and sinusitis. Some scholars have proven that this method can effectively restore the function of the Eustachian tube in children. A variety of methods have been used to assess Eustachian tube function, including the clinically used tympanogram, TMM, and ETDQ-7 scoring.^22^, ^23^, ^24^ However, studies have shown that these methods have their own advantages and disadvantages, and none of them can be recommended as a separate diagnostic criterion for Eustachian tube dysfunction.^25^ In the present study, in addition to assessing Eustachian tube function using a combination of objective and subjective methods, i.e., by the T-ETDQ scale, which is a combination of the TMM and the ETDQ-7. The results of this study showed that the T-ETDQ scores of the observation group were lower than those of the control group, suggesting that MFNS treatment can effectively improve the function of the Eustachian tube in children, which is mainly due to the fact that MFNS can control inflammation of the mucosa of the nasopharynx, eliminate swelling of the mucosa, reduce inflammation and swelling of the nasopharynx caused by the mechanical compression of the Eustachian tube, prompting the opening of the Eustachian tube to alleviate the negative pressure in the middle ear cavity, so that the middle ear exudate is reduced or discharged, which in turn improves the function of the Eustachian tube.

This study found that the safety of MFNS for the treatment of SOM in younger children is higher, which is mainly because the long-term use of MFNS does not affect the adrenal cortex function. After nasal use, the systemic concentration of the drug is low, and its post-absorption bioavailability is also low, so the inhibitory effect on the hypothalamus-pituitary-adrenal axis is small and the incidence of adverse reactions in children is low.^26^

This study has some limitations, the authors only considered the effect of treatment on children with SOM in terms of hearing and tympanic chamber, and did not explore more related to the mechanism of treatment. It is known that MFNS can affect the synthesis and release of inflammatory mediators and nasopharyngeal inflammatory response. In addition, considering the children's co-operation with some tests, only some tests with easier co-operation were used for evaluation in this study. In subsequent studies, it is necessary to explore the inflammation-related factors in serum or secretions of children with SOM treated with nasal washings combined with MFNS in order to assess the immune profile of the children at the time of recovery.

In conclusion, the clinical efficacy of MFNS in the treatment of SOM in younger children is high, which can improve the clinical symptoms of the children, promote the recovery of hearing and Eustachian tube function, reduce the local inflammatory reaction, and improve the immune function of the body. However, since children under 5 years of age have a high incidence of SOM and are in a critical period of language development, in the follow-up study, the authors will continue to focus on children at this age.

## Authors’ contributions

Xiaoyan Yu designed the research study. Lang Xu, Youqi Xie and Mengjie Huang performed the research. Xiaoyan Yu and Mengjie Huang provided help and advice on the experiments. Lang Xu and Youqi Xie analyzed the data. Xiaoyan Yu wrote the manuscript. Xiaoyan Yu and Mengjie Huang reviewed and edited the manuscript. All authors contributed to editorial changes in the manuscript. All authors read and approved the final manuscript.

## Availability of data and materials

The datasets used and/or analyzed during the present study are available from the corresponding author on reasonable request.

## Ethics approval and consent to participate

The present study was approved by the Ethics Committee of Chengdu Women's and Children's Central Hospital and written informed consent was provided by all patients prior to the study start (approval number: 202003CD28). All procedures were performed in accordance with the ethical standards of the Institutional Review Board and The Declaration of Helsinki, and its later amendments or comparable ethical standards.

## Funding

Not applicable.

## Conflicts of interest

The authors declare no conflicts of interest.
